# From Adversity to Purpose: How Self-compassion and Age Influence Meaning in Life Among Adults with Adverse Childhood Experiences

**DOI:** 10.1007/s10804-025-09547-5

**Published:** 2025-12-06

**Authors:** Miya M. Gentry, Deepa Manjanatha, Molly A. Patapoff, Barton W. Palmer

**Affiliations:** 1 San Diego State University/University of California San Diego Joint Doctoral Program in Clinical Psychology, 6363 Alvarado Court, San Diego, CA USA; 2 Sam and Rose Stein Institute for Research on Aging/Center for Healthy Aging, University of California, La Jolla, San Diego, CA USA; 3 Department of Psychiatry, University of California San Diego, La Jolla, USA; 4 Sanford Center for Empathy and Compassion, UC San Diego Health, San Diego, USA

**Keywords:** Adverse childhood experiences, Meaning in life, Self-compassion, Resilience, Lifespan development

## Abstract

Adverse childhood experiences (ACEs) are linked to negative health outcomes across the lifespan but less is known about psychological resources that might mitigate these effects. Meaning in life, conceptualized as presence (i.e., a sense of purpose and coherence) and search (i.e., striving for meaning), and self-compassion may serve as resilience factors that buffer the long-term impact of early adversity. The present study examined associations among ACEs, self-compassion, age, and meaning in life, testing whether self-compassion and age moderated the effects of ACEs. Participants were 875 community-dwelling adults (M = 66.1, SD = 20.8). Multiple linear regression models tested main and interaction effects of age and self-compassion on outcomes of presence of and search for meaning in life. Results indicated that self-compassion was a robust positive predictor of presence of meaning and a negative predictor in search for meaning. ACEs predicted lower presence of meaning, and both self-compassion and age moderated this relationship, such that adverse effects of ACEs were attenuated among individuals higher in self-compassion and among older adults. For search, self-compassion predicted lower search for meaning, while ACEs and the moderation effect were not statistically significant. These findings underscore the importance of distinguishing presence and search as distinct constructs and highlights the protective role of psychological and developmental resources in meaning-making following adversity. Self-compassion may represent a promising target for interventions aimed at fostering resilience in individuals with ACEs.

## Introduction

Adverse childhood experiences (ACEs) encompass various forms of abuse, neglect, and household dysfunction and represent a significant public health concern due to their pervasive and lasting effects (Negriff, 2020). Approximately 64% of U.S. adults report experiencing at least one ACE, and 17% report exposure to four or more (Swedo, 2023). These ACEs negatively impact physical, psychological, and social health across the lifespan, contributing to chronic medical conditions and psychiatric disorders, and can perpetuate intergenerational cycles of trauma and adversity (Negriff, 2020; Noll et al., 2009). While the extensive focus on these deleterious outcomes underscores the importance of preventing ACEs, there is also a clear need to examine factors that might mitigate these harmful, long-term effects. A promising avenue in the latter vein is the exploration of positive psychological constructs that may foster adaptation and well-being in the face of early adversity (Jeste et al., 2015; Seligman & Csikszentmihalyi, 2014). Among various positive psychological factors, two potentially modifiable ones that may have relevance for such adaptation and well-being in this population are meaning in life and self-compassion (Neff, 2003; Park, 2010; Southwick et al., 2014).

Meaning in life, a critical component of well-being, encompasses two dimensions: the presence of meaning (i.e., a sense of purpose and coherence) and the search for meaning (i.e., active efforts to find significance) in life (Steger et al., 2006). These dimensions play distinct roles in psychological health; the presence of meaning is linked to greater life satisfaction, while the search for meaning can reflect either adaptive growth or distress depending on context (Aftab et al., 2019). In broader trauma literature, theoretical models, such as the meaning-making model, suggest that meaning-making after adversity serves as both a coping mechanism and an indicator of long-term psychological adaptation following trauma exposure (Park, 2010, 2022). Over time, individuals may come to accept past adversities by recognizing how these experiences fostered personal growth, which may enable them to find positive meaning in aspects of their lives. Consistent with this model, research has shown that individuals with a stable sense of meaning report greater physical and mental health, while those actively seeking meaning may experience heightened emotional distress in certain contexts (Martela & Steger, 2016; Rose et al., 2023).

Complementing the role of meaning in life, self-compassion, the practice of extending kindness and understanding to oneself during adversity, is a key resilience factor for individuals with trauma histories (Neff, 2003). It buffers against the psychological impacts of ACEs, enhances coping, and supports meaning-making processes (Finlay-Jones et al., 2023; Germer & Neff, 2013). Within the context of the resilience framework, self-compassion and meaning in life are interconnected pathways that support adaptation and recovery following adversity (Southwick et al., 2014). Self-compassion may mitigate emotional distress, foster the integration of adverse experiences, and promote coherent life narratives that reinforce meaning. This interplay appears to evolve with age, as research suggests self-compassion often increases in adulthood and may be associated with greater meaning in life among older adults (Phillips & Ferguson, 2013). However, mixed findings on the role of age (Stapleton et al., 2018) underscore the need for further exploration into how self-compassion and age shape the relationship between ACEs and meaning in life.

Age is a significant factor in the balance between the search for and the presence of meaning in life. Older individuals tend to report higher levels of presence of meaning and lower levels of search for meaning compared to younger adults, reflecting developmental and contextual influences such as the cumulative impact of life experiences and shifting priorities with age (Aftab et al., 2019; Steger et al., 2009). Similarly, self-compassion generally increases in adulthood (Allen et al., 2012; Murn & Steele, 2020), though findings among older adults remain mixed; for example, one study found a weak, non-significant correlation between self-compassion and age in adults over 65, suggesting complexity in this relationship (Phillips & Ferguson, 2013). These lifespan variations in self-compassion may influence age-related differences in meaning-making, particularly in reconciling past ACEs to foster a clearer sense of purpose. Despite the importance of ACEs, self-compassion, and age, little is known about how these factors collectively shape meaning in life during adulthood, and research directly addressing these associations is limited (Rose et al., 2023; Weibel et al., 2017).

The present study examined the relationships among ACEs, age, self-compassion, and meaning in life, with a focus on how self-compassion and age shape these associations. Grounded in the meaning-making model (Park, 2010, 2022) and resilience framework (Southwick et al., 2014), we hypothesized that greater ACE exposure would be associated with lower presence and higher search for meaning. We further hypothesized that self-compassion would buffer these associations, such that individuals with higher self-compassion would show greater presence and lower search despite higher ACEs. Age was included as an exploratory moderator to examine whether it influenced the strength or direction of these relationships. Given evidence suggesting that meaning-making and resilience processes may vary across sociocultural contexts (Lewis et al., 2015; Williams et al., 2019), race/ethnicity was included as a statistical covariate to adjust for potential socio-demographic variability.

## Methods

### Participants and Procedure

Data for the current report were collected as part of the Successful AGing Evaluation (SAGE) study conducted by the Sam and Rose Stein Institute for Research on Aging/Center for Healthy Aging at the University of California San Diego (UC San Diego). The SAGE study was initiated in 2010 as a longitudinal survey of an age-stratified sample of adults in the San Diego County region (see: Aftab et al., 2019; Jeste et al., 2013). Due to the particulars of when questionnaires of the key constructs for the present analyses were included in the larger SAGE survey, the current analyses are based on cross-sectional data collected between January 2014 and November 2016. Inclusion criteria for a larger SAGE study were (1) aged 21 years and older, (2) physical and mental ability to participate in a telephone interview and to complete an online or paper-and-pencil mail survey, (3) informed consent for study participation, and (4) English fluency. Participants were excluded if they resided in a nursing home or needed daily skilled nursing care, reported a formal diagnosis of dementia as made by a clinician, had a terminal illness, or needed hospice care. For inclusion in the present analyses, we also required completed data for three measures (described in more detail below): the Adverse Childhood Experiences Questionnaire, the Meaning in Life Questionnaire, and the Self-Compassion Scale. The SAGE study was approved by the UC San Diego Office of IRB Administration at UC San Diego.

The final sample included 875 community-dwelling adults. Mean (SD) age was 66.1 (20.8) years, and education was 15.4 (2.2) years. Gender distribution was approximately equal (50.6% women and 49.4% men). In terms of race/ethnic background, the sample included 674 (77.0%) non-Latine White, 120 (13.8%) Latine, 57 (6.5%) Asian, 12 (1.4%) Black, 3 (0.3%) Indigenous American, and 6 (0.7%) people of other backgrounds.

### Measures

All measures were based on self-report questionnaires administered via standard mail or web-based format.

#### Sociodemographic Characteristics

Sociodemographic information included age at the time of collection of data for this report, gender, education, and race/ethnicity.

#### Adverse Childhood Experiences

The Adverse Childhood Experiences Questionnaire (ACEs) is a 10-item, self-report retrospective questionnaire (Felitti et al., 1998). The questionnaire assesses exposure to 10 types of intrafamilial adverse experiences that belong to two categories: maltreatment (i.e., physical abuse, emotional abuse, sexual abuse, physical neglect, emotional neglect) and household dysfunction (i.e., parental separation/divorce, household physical violence, household substance abuse, household mental illness or suicide attempt, incarcerated household member). All items are answered either “yes” or “no”. Based on the number of ACE endorsed “yes” the total score has a potential range of 0 to 10. The ACE questionnaire has high internal consistency (coefficient alpha = 0.70–0.90; Felitti et al., 1998).

#### Meaning in Life

The Meaning in Life Questionnaire (MLQ) is a 10-item questionnaire (Steger et al., 2006), and has become the most widely used measure in research on meaning in life (King & Hicks, 2021). Two five-item subscales measure one’s sense of current search for meaning and presence of meaning in life. Each item is rated from 1 (*Absolutely Untrue*) to 7 (*Absolutely True*) by respondents, yielding a potential range of 5 to 35 points for each subscale. The two-factor structure has been replicated multiple times, and both subscales have good internal consistency (coefficient alpha = 0.82–0.88; King & Hicks, 2021).

#### Self-compassion

The Self-Compassion Scale (SCS) is currently the most widely used self-compassion measure (Neff, 2003). Although originally developed as a 26-item questionnaire, the 12-item short form (SCS-SF; Raes et al., 2011) employed in the current study has shown strong internal consistency (coefficient alpha = 0.85–0.90). The SCS-SF has a six-factor structure which includes: self-kindness, self-judgment, common humanity, isolation, mindfulness, and over-identification. Each item is rated on a 5-point Likert scale ranging from 1 (*Almost Never*) to 5 (*Almost Always*). Scores for negative subscales are reverse-scored, and an overall self-compassion score is calculated as having a potential range of 12 to 60, with higher scores reflecting more self-compassion.

### Statistical Analyses

All analyses were conducted using IBM SPSS Statistics Software (Version 28). Descriptive statistics included means, standard deviations, and proportions. For inferential statistics, multiple linear regression analyses were used to test the effects of ACEs, age, and self-compassion on two outcome variables: the presence of meaning and the search for meaning in life. Covariates of gender (coded as 0 = women and 1 = men) and race/ethnicity (recoded as a dichotomous variable: 0 = White and 1 = non-White) were included in all models.

Prior to analyses, age, ACEs, and self-compassion were each mean-centered to facilitate the interpretation of interaction terms. To examine two-way interactions, we created interaction terms by multiplying the centered ACEs score by centered self-compassion and separately multiplying the centered ACEs score by centered age. The strength of associations and effect sizes (i.e., “small” = 0.10–0.29, “medium” = 0.30–0.49, “large” = 0.50 or greater) were evaluated using standardized beta coefficients (β; Nieminen, 2022), and significance was determined with a threshold of p < .05 (two-tailed)**.** Confidence intervals (95% CIs) were reported for all significant findings and used to determine the statistical significance of the interaction effects (Cohen, 1994; Hayes, 2017).

## Results

### Descriptive Statistics

Fifty-two percent (*n* = 486) of participants endorsed experiencing at least one ACE. The two highest endorsed ACEs were living with someone who has been incarcerated (21.8%, *n* = 187) and physical abuse (21.6%, *n* = 117). The lowest endorsed ACE was child sexual abuse (4.0%,* n* = 34). The mean score for ACEs was 1.28 (*SD* = 1.71), indicating a low overall reported childhood adversity in the sample. The mean self-compassion score was 42.14 (*SD* = 7.63), reflecting moderate levels of self-compassion among participants.

Participants had a mean MLQ Presence subscale score of 26.78 (*SD* = 5.90), suggesting high perceived meaning in life. On the MLQ Search subscale, the mean score was 18.44 (*SD* = 7.96), suggesting a moderate degree to which participants were actively searching for meaning in life.

### Presence of Meaning

#### Moderation of Self-compassion by ACEs

**Fig. 1:**
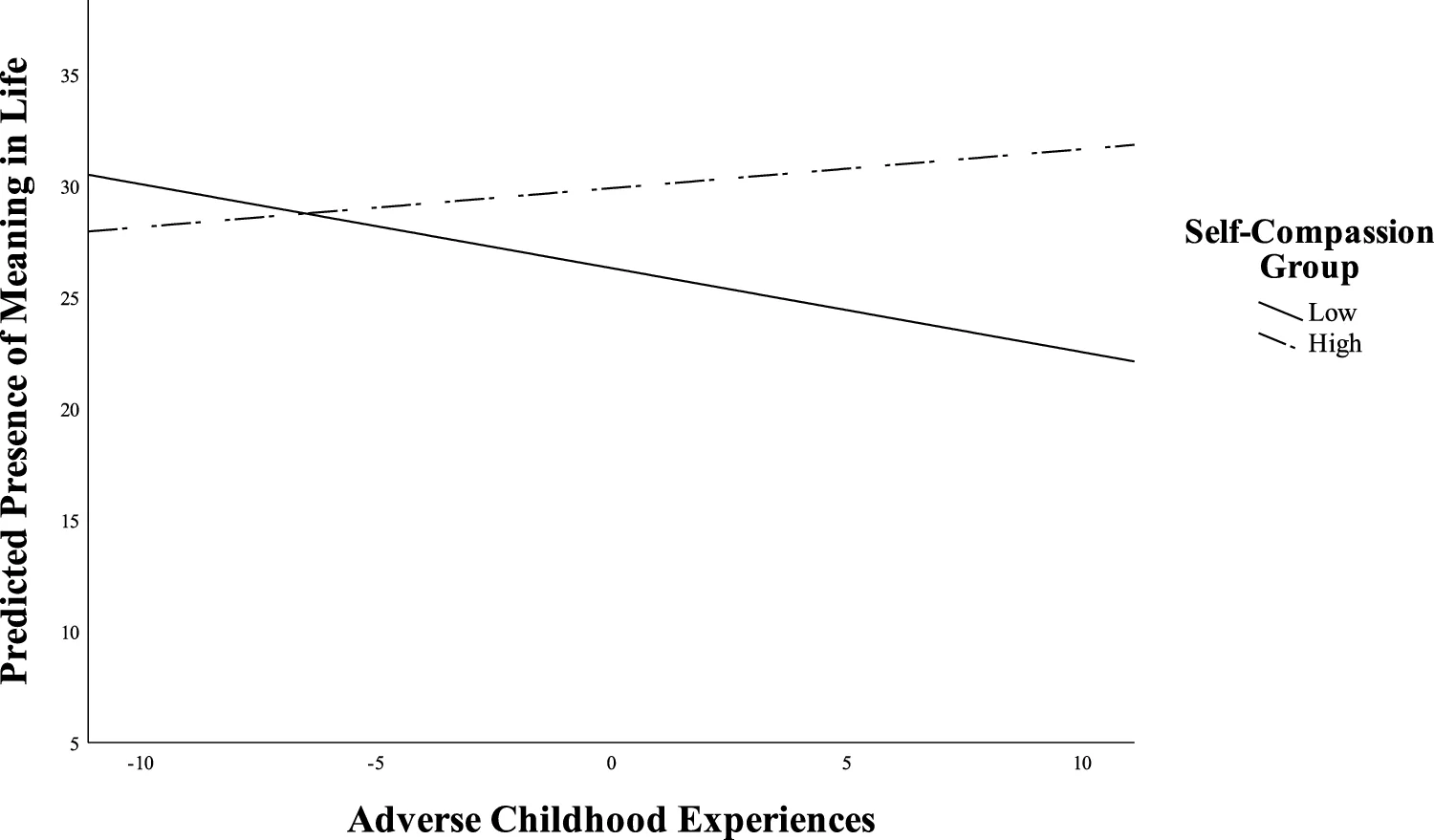
Interaction of adverse childhood experiences and self-compassion predicting presence of meaning in life. *Predicted values of presence of meaning in life are plotted at low (−1 SD) and high (+ 1 SD) levels of self-compassion. Higher scores indicate greater presence of meaning. ACEs were mean-centered; negative values on the x-axis represent scores below the sample mean

All coefficients and respective test statistics for models focused on self-compassion can be found on Table 1. The overall model, including the ACEs and self-compassion interaction term, was significant [*F*(5, 753) = 25.78, *p* < 0.001], accounting for 14.6% of variance in the presence of meaning for life (*R*^2^ = .146). Self-compassion was a robust positive predictor with a medium effect size (β = .34, *p* < 0.001), such that higher self-compassion was associated with greater presence of meaning. Gender was also significant (β = −.11, *p* = 0.002), with men reporting lower presence of meaning compared to women. Race/ethnicity emerged as a small positive effect (β = .07, *p* = 0.031), with non-White participants reporting greater presence relative to White participants. Importantly, the interaction term of ACEs by Self-Compassion was statistically significant (β = .10, *p* = 0.005). This interaction is depicted in Fig. 1. The main effect of ACEs was not statistically significant.

**Table 1:** Moderation of ACEs by self-compassion predicting presence of meaning

Variable	β	SE	*t*	*p*	95% CI [LL, UL]
**Gender**	**−.106**	**0.404**	**−3.107**	**0.002**	**[−2.050, −0.463]**
**Race/ethnicity**	**.073**	**0.484**	**2.163**	**0.031**	**[0.097, 1.998]**
ACEs	−.046	0.122	−1.329	0.184	[−0.403, 0.077]
**Self-compassion (SC)**	**.336**	**0.027**	**9.805**	** < 0.001**	**[0.208, 0.312]**
ACEs*SC	**.097**	**0.015**	**2.808**	**0.005**	**[0.012, 0.070]**

*ACE = Adverse Childhood Experiences. Variables of ACEs and self-compassion have been mean-centered for interpretability. Gender is coded as 0 = women, 1 = men. Race/ethnicity was coded as a dichotomous variable (0 = non-Latine White, 1 = non-White [Black/African-American, Latine, Asian, Indigenous American, Pacific Islander/Native Hawaiian, multi-racial, and “Other”]). Bolded coefficients and test statistics represent a two-tailed significance of < 0.05

#### Moderation of Age by ACEs

**Fig. 2:**
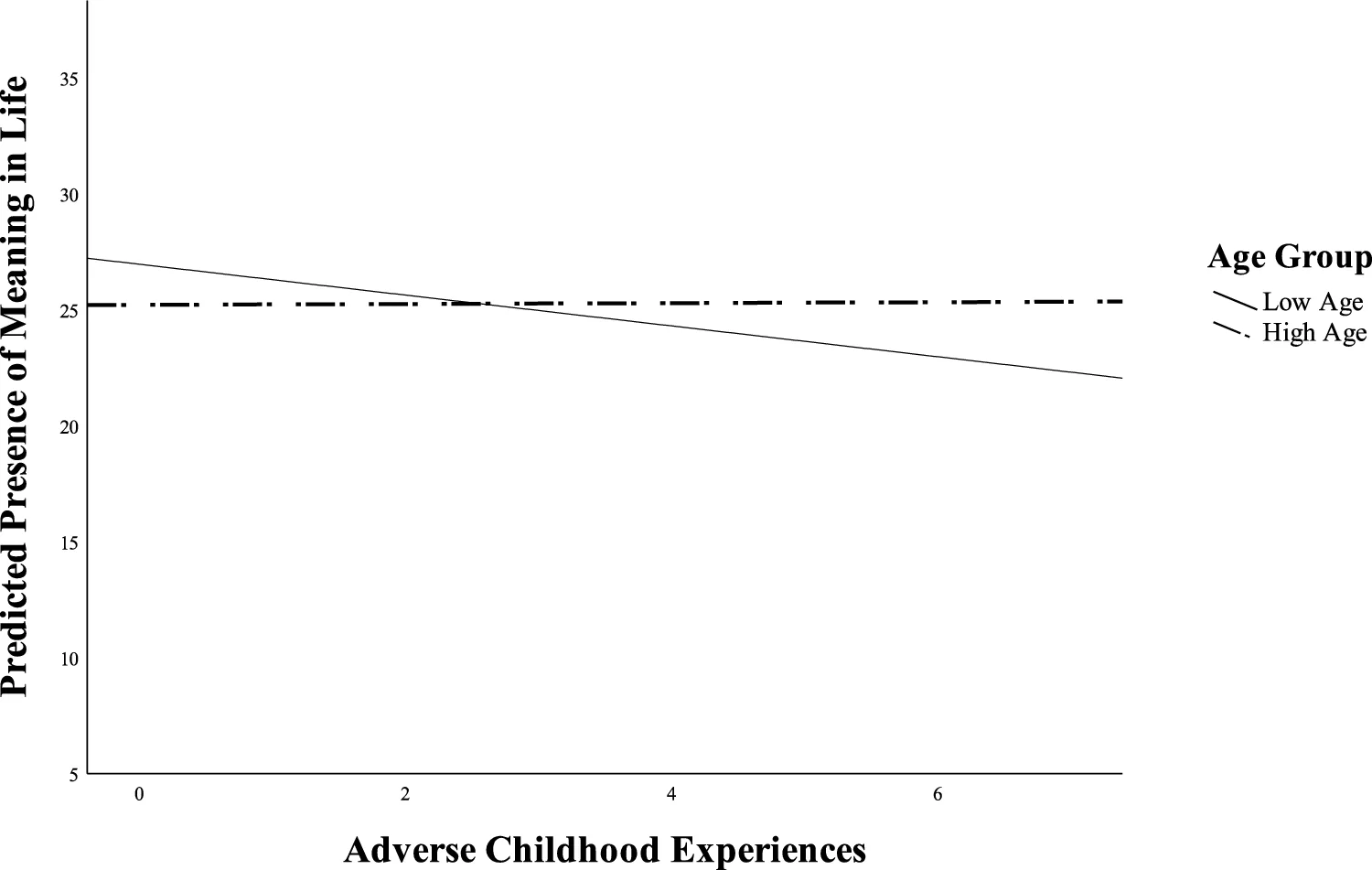
Interaction between adverse childhood experiences (ACEs) and age predicting presence of meaning in life. *Predicted values of presence of meaning in life are plotted for low (−1 SD) and high (+ 1 SD) age. ACEs were mean-centered; values on the x-axis reflect centered scores

All coefficients and respective test statistics for models focused on age are provided in Table 2. The overall model, including the ACEs and age interaction term, was significant [*F*(5, 792) = 4.50, *p* < 0.001], accounting for 2.8% of variance in the presence of meaning for life (*R*^*2*^ = 0.028). Significant main effects were observed for gender, race/ethnicity, and ACEs. Gender emerged as a significant predictor (β = −.09, *p* = 0.017), with men reporting lower presence of meaning compared to women. Race/ethnicity also showed a small positive effect (β = .09, *p* = 0.030), with non-White participants reporting slightly greater presence relative to White participants. ACEs were a negative predictor of presence of meaning (β = −.08, *p* = 0.032), indicating that individuals reporting more childhood adversity endorsed lower meaning in life. The main effect of age was not statistically significant (β = −.01, *p* = 0.710), falling below the threshold for a small effect (|β|< 0.10) (Cohen, 1994). Importantly, the interaction term was statistically significant, (β = −.09, *p* = 0.015), indicating that the negative association between ACEs and presence of meaning was weaker among older adults, such that even at higher levels of ACEs, older adults continued to report relatively high levels of meaning in life. This interaction is depicted in Fig. 2. Although the effect size was small, the interaction was statistically reliable, as indicated by regression coefficients, confidence intervals, and p-values.

**Table 2:** Moderation of ACEs by age predicting presence of meaning

Variable	β	SE	*t*	*p*	95% CI [LL, UL]
**Gender**	**−.085**	**0.422**	**−2.386**	**0.017**	**[−1.833, −0.178]**
**Race/ethnicity**	**.077**	**0.510**	**2.176**	**0.030**	**[−0.109, 2.113]**
**ACEs**	**−.080**	**0.131**	**−2.144**	**0.032**	**[−0.540, −0.024]**
Age	−.013	0.010	−0.371	0.710	[−0.024, 0.017]
ACEs*age	**.089**	**0.007**	**2.438**	**0.015**	**[0.003, 0.029]**

*ACE = Adverse Childhood Experiences. Variables of ACEs and age have been mean-centered for interpretability. Gender is coded as 0 = women, 1 = men. Race/ethnicity was coded as a dichotomous variable (0 = non-Latine White, 1 = non-White [Black/African-American, Latine, Asian, Indigenous American, Pacific Islander/Native Hawaiian, multi-racial, and “Other”]). Bolded coefficients and test statistics represent a two-tailed significance of < 0.05

### Search for Meaning

#### Moderation of Self-compassion by ACEs

All coefficients and respective test statistics for models focused on self-compassion can be found on Table 3. The overall model, including the ACEs and self-compassion interaction term, was significant [*F*(5, 777) = 4.64, *p* < 0.001], accounting for 2.9% of variance in the search for meaning for life (*R*^2^ = .029). Self-compassion was a robust negative predictor with a medium effect size (β = −0.23, *p* < 0.001), indicating that higher self-compassion was associated with lower search for meaning. Race/ethnicity also showed a small effect (β = .11, *p* = 0.002), with non-White participants reporting greater search relative to White participants. The main effect of ACEs and the covariate of gender were not statistically significant in this model. The interaction term between ACEs and self-compassion was not statistically significant, suggesting that self-compassion did not moderate the association between ACEs and search for meaning.

**Table 3:** Moderation of ACEs by self-compassion predicting search for meaning

Variable	β	SE	*t*	*p*	95% CI [LL, UL]
Gender	−.031	0.573	−0.861	0.390	[−1.618, −0.632]
**Race/ethnicity**	**.112**	**0.573**	**3.152**	**0.002**	**[0.821, 3.533]**
ACEs	.047	0.172	1.290	0.198	[−0.116, 0.558]
**Self-compassion**	**−.227**	**0.038**	**−6.270**	** < 0.001**	**[−0.310, −0.162]**
ACEs*self-compassion	.037	0.020	1.018	0.309	[−0.019, 0.061]

*Variables of ACEs and self-compassion have been mean-centered for interpretability. Gender is coded as 0 = women, 1 = men. Race/ethnicity was coded as a dichotomous variable (0 = non-Latine White, 1 = non-White [Black/African-American, Latine, Asian, Indigenous American, Pacific Islander/Native Hawaiian, multi-racial, and “Other”]). Bolded coefficients and test statistics represent a two-tailed significance of < 0.05

#### Moderation of Age by ACEs

All coefficients and respective test statistics for models focused on self-compassion can be found on Table 4. The overall model, including the ACEs and age interaction term, was significant [*F*(5, 777) = 4.64, *p* < 0.001], accounting for 2.9% of variance in the search for meaning for life (*R*^2^ = .029). Significant main effects were observed for race/ethnicity and age. Race/ethnicity showed a small positive effect (β = .09, *p* = 0.010), with non-White participants reporting greater search for meaning compared to White participants. Age was also a small negative predictor (β = −.09, *p* = 0.011), indicating that older adults reported lower search for meaning relative to younger adults. The main effect of ACEs and the covariate of gender were not statistically significant in this model. The interaction term between ACEs and age was not statistically significant, indicating that age did not moderate the association between ACEs and search for meaning.

**Table 4:** Moderation of ACEs by age predicting search for meaning

Variable	β	SE	*t*	*p*	95% CI [LL, UL]
Gender	−.037	0.573	−1.023	0.307	[−1.710, 0.538]
**Race/ethnicity**	**.093**	**0.696**	**2.599**	**0.010**	**[0.445, 3.189]**
ACEs	.055	0.176	1.460	0.145	[−0.088, 0.601]
**Age**	**−.094**	**0.014**	**−2.563**	**0.011**	**[−0.064, −0.009]**
ACEs*Age	−.008	0.009	−0.206	0.837	[−0.019, 0.016]

*Variables of ACEs and age have been mean-centered for interpretability. Gender is coded as 0 = women, 1 = men. Race/ethnicity was coded as a dichotomous variable (0 = non-Latine White, 1 = non-White [Black/African-American, Latine, Asian, Indigenous American, Pacific Islander/Native Hawaiian, multi-racial, and “Other”]). Bolded coefficients and test statistics represent a two-tailed significance of < 0.05

## Discussion

The present study examined how adverse childhood experiences (ACEs) self-compassion, and age shape two dimensions of meaning in life: presence and search. Our findings provided partial support for hypotheses. Consistent with our hypotheses, ACEs were associated with lower presence of meaning, and self-compassion and age moderated the association between ACEs and presence of meaning. In contrast, models predicting search for meaning revealed a different pattern: self-compassion consistently predicted lower search, while ACEs and the moderation effects were not statistically significant.

Consistent with theoretical models, self-compassion emerged as the strongest predictor of the presence of meaning, supporting the theory that adaptive ways of relating to oneself, such as self-compassion, protect against the lasting effects of adversity (Finlay-Jones et al., 2023; Neff, 2003). Self-compassion may facilitate meaning by allowing individuals to acknowledge adversity without being overwhelmed by self-criticism, fostering acceptance and coherence in one’s life narrative (Germer & Neff, 2013). Our findings coincide with Park's (2010, 2022) meaning-making framework, which suggest that psychological resources aid in integrating adverse experiences into broader belief systems and goals. Beyond self-compassion, both interaction terms (self-compassion and age) were statistically significant, highlighting two complementary pathways of resilience. First, self-compassion buffered against the negative impact of ACEs on presence of meaning in life, consistent with resilience frameworks that emphasize adaptive coping resources in response to stress (Southwick et al., 2014). Second, older adults reported higher presence of meaning under conditions of greater adversity compared to younger adults, suggesting developmental shifts in meaning-making across the lifespan (Steger et al., 2009). With age, accumulated life experience may provide perspective that enables individuals to contextualize early adversity within broader life narratives, a process aligned with lifespan theories of meaning-making and well-being (Aftab et al., 2019). Taken together, these findings may suggest that both psychological (i.e., self-compassion) and developmental (i.e., age) resources can mitigate the erosive effects of ACEs on meaning in life.

A different pattern emerged for the search for meaning. Across all models, self-compassion was a robust negative predictor, suggesting that individuals higher in self-compassion reported less active searching for meaning. This finding fits with prior work conceptualizing search as ambivalent, sometimes reflecting growth-oriented striving, but often reflecting existential struggle (Aftab et al., 2019; Steger et al., 2006). The protective role of self-compassion may lie in reducing the distress-driven aspects of search by offering a stable sense of acceptance and coherence (Neff, 2003). Whereas self-compassion emerged as a robust predictor of search for meaning, ACEs were not directly associated with search, echoing prior findings that adversity may more strongly influence the dimensions of coherence or significance rather the search dimension (Rose et al., 2023).

Our findings also suggest that age and race/ethnicity may affect search for meaning in life. Specifically, age was negatively associated with search, consistent with lifespan perspectives that emphasize a developmental decline in existential striving as priorities consolidate and coherence increases over time (Steger et al., 2009). The observed effects of race/ethnicity indicated that, on average, non-White participants reported higher search for meaning than non-Latine White participants. This may highlight the need to situate meaning-making within cultural frameworks, where differing traditions, community supports, and collective identities may shape how individuals engage with existential questions. Alternatively, these differences may also reflect differential exposure to systemic stressors such as racism and discrimination, which are well-documented predictors of psychological distress and meaning-making challenges (Lewis et al., 2015; Williams et al., 2019). Together, these findings suggest that that meaning making processes are not uniform, rather shaped by sociocultural context.

Taken together, our findings reinforce the importance of distinguishing presence and search as theoretically distinct but related constructs (Martela & Steger, 2016). Presence appears closely tied to resilience processes, supported by both psychological and developmental factors, while search may represent a more complex and context-dependent process, less directly linked to adversity. Importantly, our small but significant moderation effects underscore the idea that resilience is multifaceted: individual capacities like self-compassion interact with developmental state to shape how adversity is integrated into meaning systems (Park, 2010; Southwick et al., 2014).

From an applied perspective, these results underscore the translational potential of self-compassion as a therapeutic target. Interventions such as Compassion-Focused Therapy (Braehler & Neff, 2020) and mindfulness-based approaches (Bluth et al., 2018) may help foster protective meaning-making processes. By integrating meaning-centered strategies (e.g., narrative reconstruction, values clarification) (Breitbart et al., 2012) alongside self-compassion training, individuals with histories of ACEs to more effectively build purpose in life. Clinically, this suggests that interventions targeting self-compassion could simultaneously reduce maladaptive search for meaning and promote the presence of meaning, ultimately enhancing psychological well-being. Preventively, embedding such strategies in community or school-based programs may bolster resilience before the negative consequences of early adversity fully emerge.

## Limitations and Future Directions

The findings from the current study need to be considered in light of limitations. First, due to cross-sectional correlational analyses, we cannot infer any causality in the relationships between ACEs, self-compassion, age, or meaning in life. Future longitudinal and experimental studies are needed to examine how these relationships unfold over time and whether self-compassion interventions can directly influence trajectories of meaning-making. Second, ACEs were assessed via retrospective self-report, which introduces potential recall bias or underreporting and could affect the pattern of findings. Longitudinal studies following individuals from childhood to adulthood are needed for more definitive conclusions. Nonetheless, the present findings remain informative, as carrying conscious memories of adversity appears linked to reduced sense of meaning, a process self-compassion may help buffer. Third, the average ACEs score in the sample was relatively low (mean = 1.28, SD = 1.71), compared to population-level estimates where approximately 61% of adults report at least one ACE (Swedo, 2023). This difference may reflect the demographic characteristics of our sample, such as the underrepresentation of individuals with higher ACE scores and an unequal age distribution, with 75% of participants being over the age of 50. However, the findings remain valuable in demonstrating how meaning in life and self-compassion function as resilience factors in a community-based sample, providing a foundation for future research on individuals with higher ACE scores or those in clinical populations. As a result, the observed effects may not fully capture the impact of severe or cumulative adversity on meaning in life. Next, although race/ethnicity emerged as a significant predictor of search for meaning, the current study was not designed to deeply examine the cultural context. The small effect observed suggests that cultural or contextual factors may shape meaning-related processes, but further investigation, with a sampling plan appropriate to testing such hypotheses, is needed to clarify how cultural identity, community resources, systemic stressors such as racism and discrimination, and lived experiences interact with adversity and resilience factors. In addition, although interaction effects for presence of meaning were statistically significant, their effect sizes were small, raising questions about practical significance. Future research should examine whether these patterns replicate in larger, more diverse samples and explore conditions under which these moderation effects are more pronounced. Finally, the current study did not account for other psychological and contextual resilience factors (e.g., optimism, social support, current stress levels), which may interact with self-compassion and age in shaping meaning-making. Including these variables in future longitudinal research could clarify the broader constellation of protective processes that promote meaning-making following early adversity.

In sum, with the above caveats in mind, the current study demonstrates that self-compassion and age uniquely contribute to meaning in life among individuals with ACE histories. Self-compassion consistently predicted greater presence and lower search, while both self-compassion and age moderated the association between ACEs and presence. These results highlight the value of distinguishing between presence of and search for meaning in life and underscore the importance of psychological and developmental resources in fostering resilience and meaning-making after early adversity.

## Data Availability

The data that support the findings of this study are not the property of the authors but were used by permission from the Sam and Rose Stein Institute for Research on Aging at the University of California San Diego. Therefore, permission to release the data to third parties is not under the discretion of the study authors but instead must be approved by the UC San Diego Stein Institute and the PI of the Successful Aging (SAGE) survey study.
